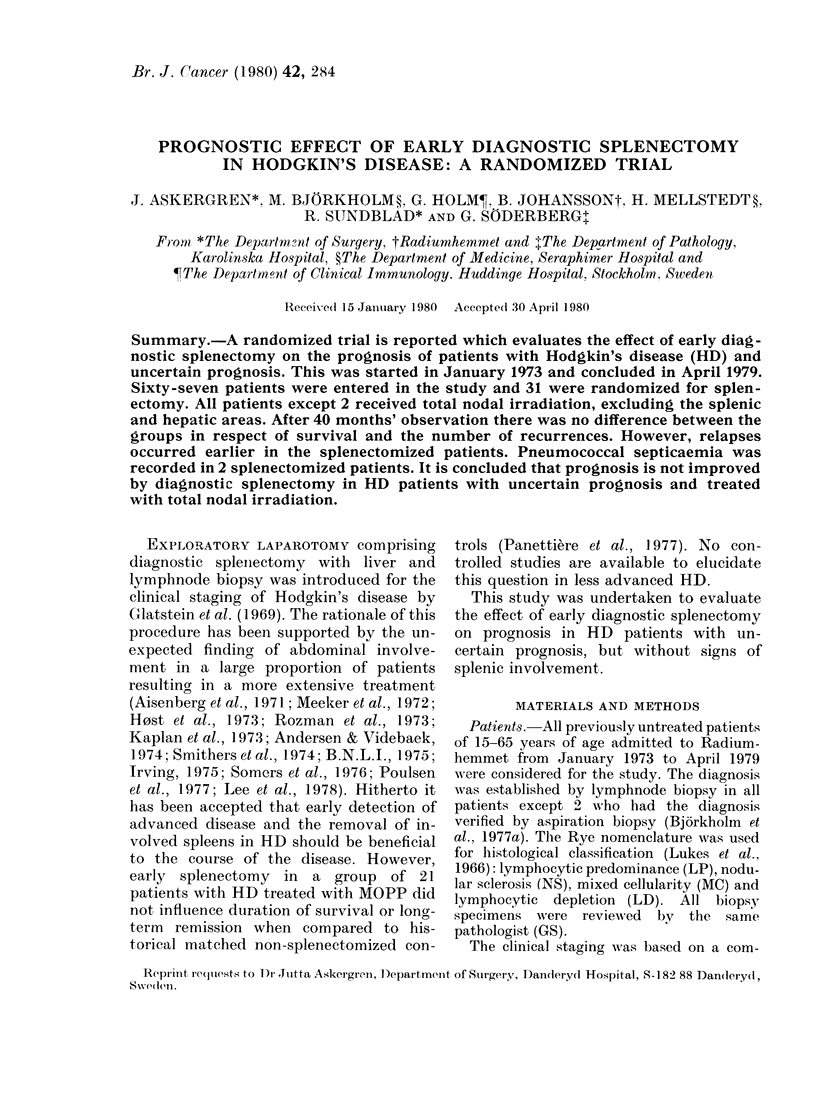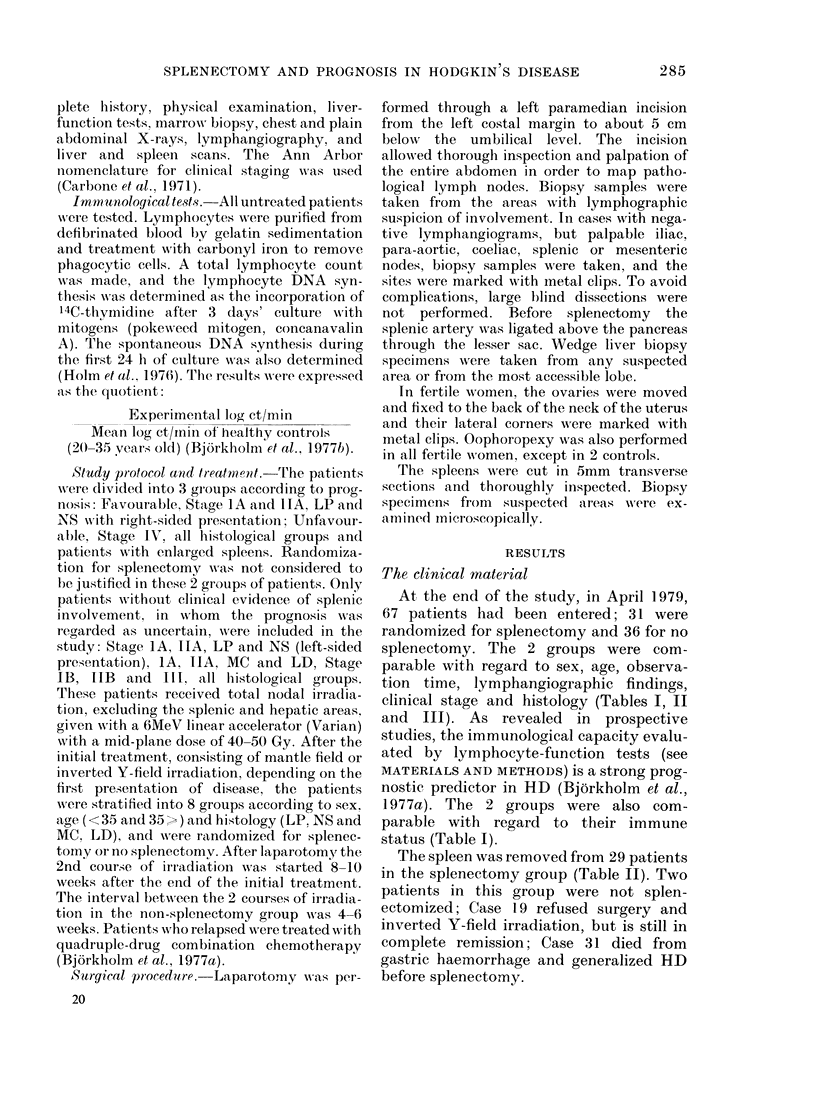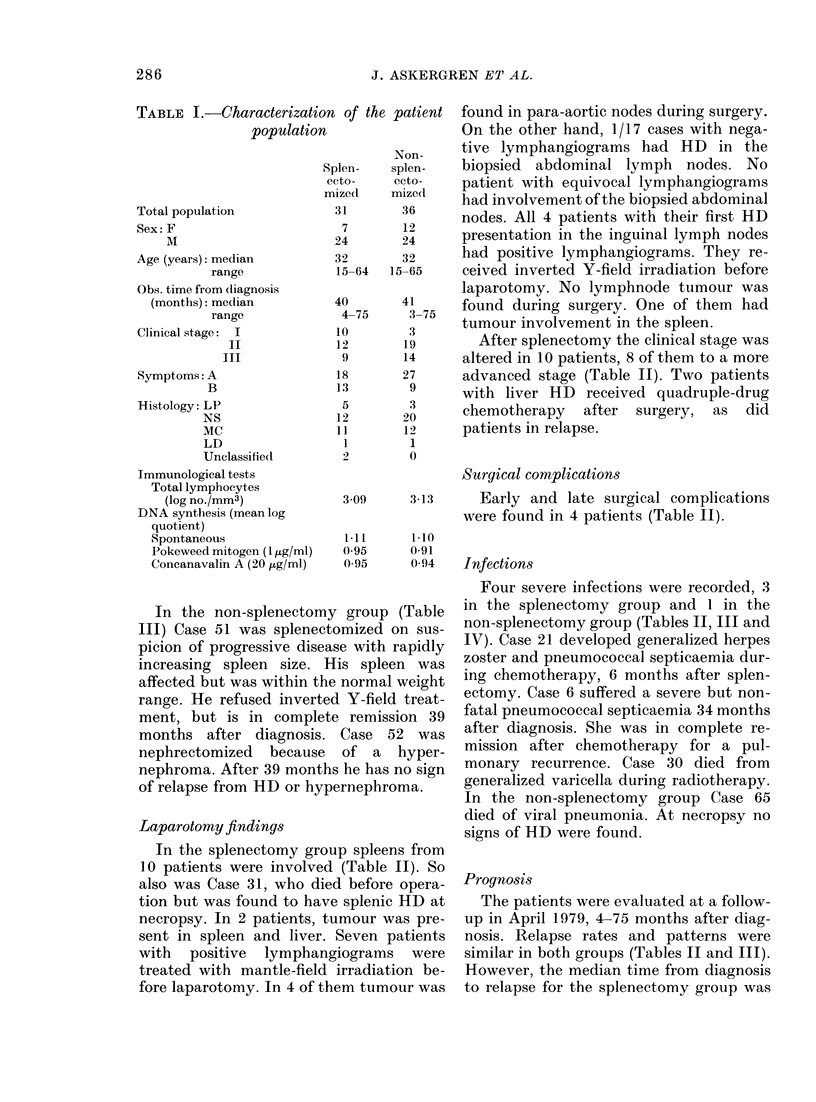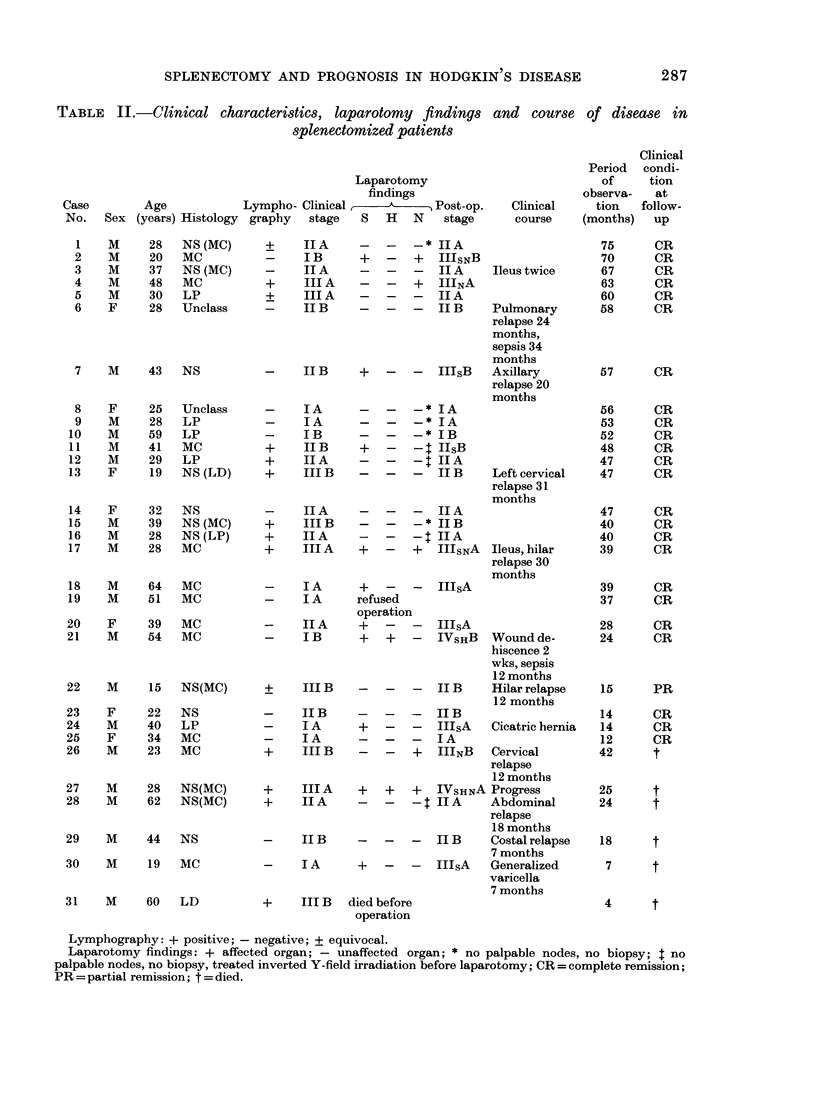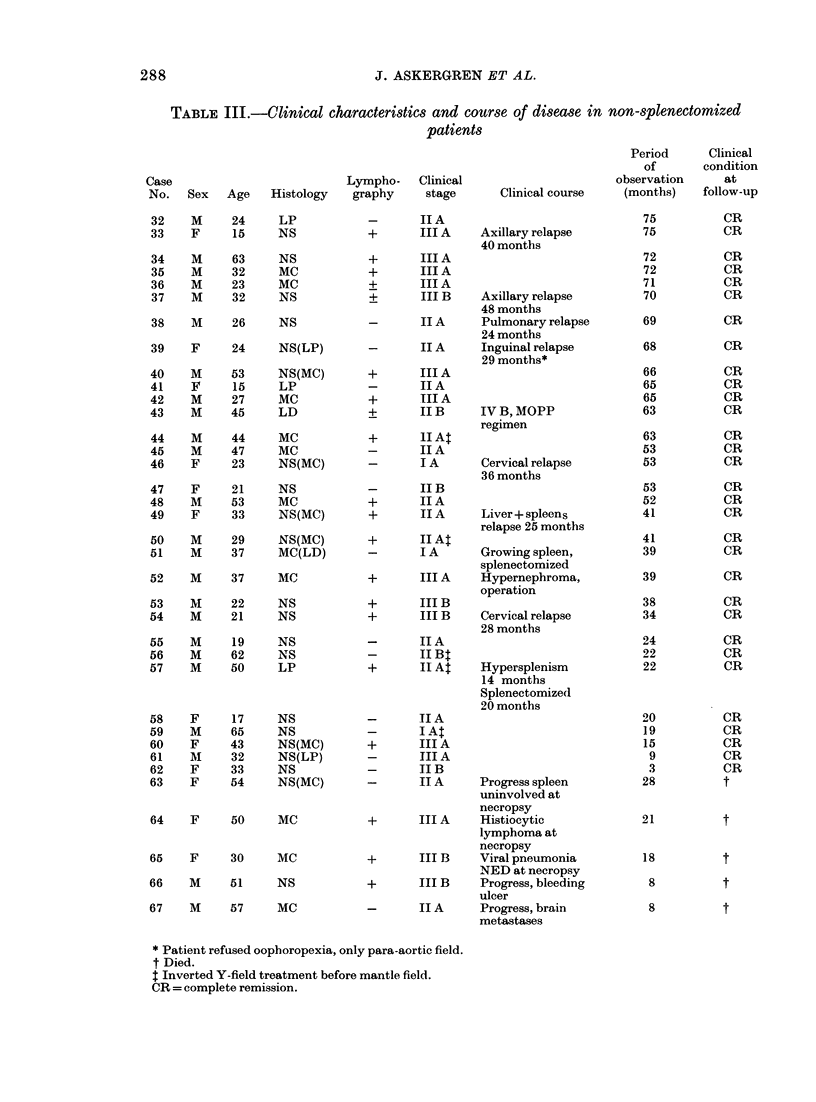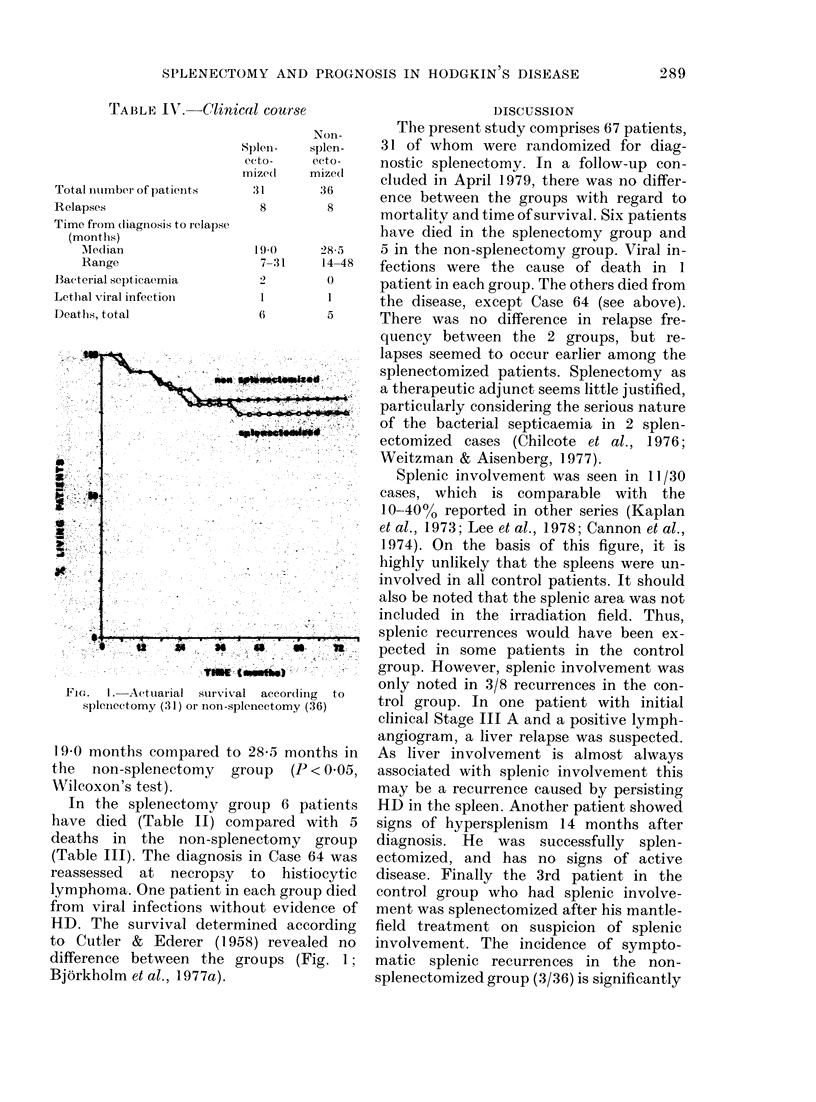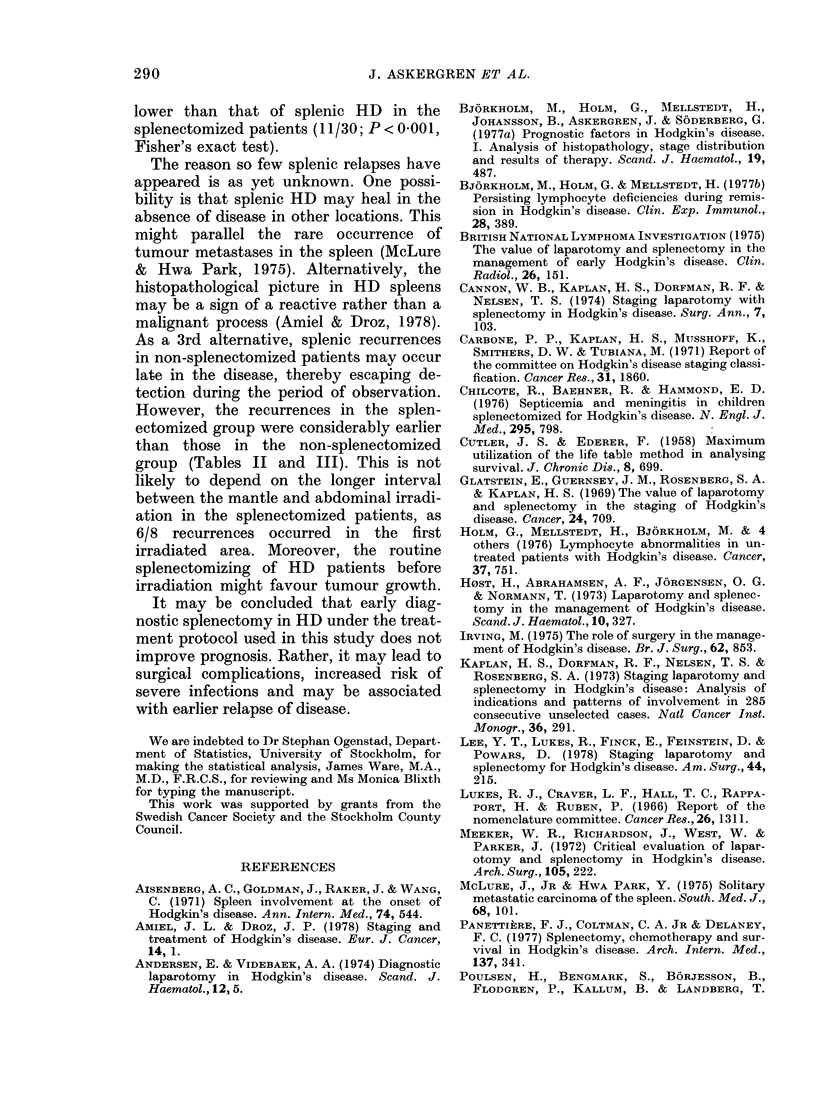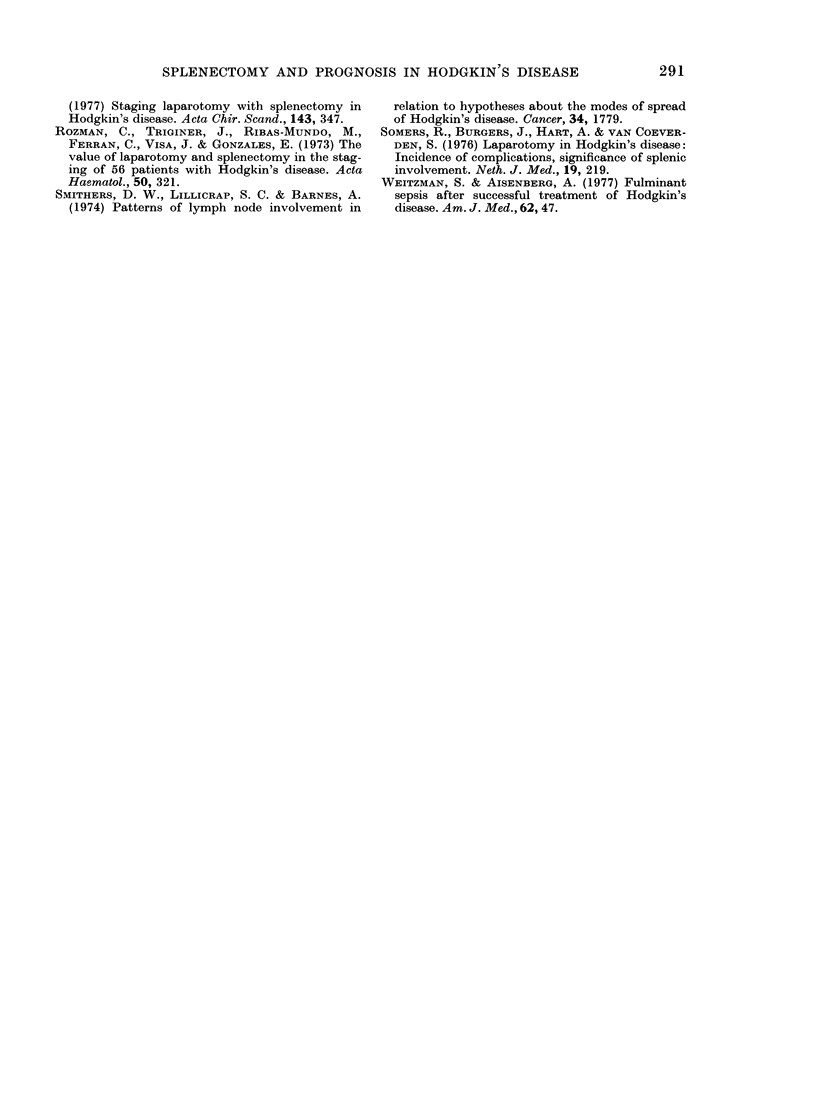# Prognostic effect of early diagnostic splenectomy in Hodgkin's disease: a randomized trial.

**DOI:** 10.1038/bjc.1980.228

**Published:** 1980-08

**Authors:** J. Askergren, M. Björkholm, G. Holm, B. Johansson, H. Mellstedt, R. Sundblad, G. Söderberg

## Abstract

A randomized trial is reported which evaluates the effect of early diagnostic splenectomy on the prognosis of patients with Hodgkin's disease (HD) and uncertain prognosis. This was started in January 1973 and concluded in April 1979. Sixty-seven patients were entered in the study and 31 were randomized for splenectomy. All patients except 2 received total nodal irradiation, excluding the splenic and hepatic areas. After 40 months' observation there was no difference between the groups in respect of survival and the number of recurrences. However, relapses occurred earlier in the splenectomized patients. Pneumococcal septicaemia was recorded in 2 splenectomized patients. It is concluded that prognosis is not improved by diagnostic splenectomy in HD patients with uncertain prognosis and treated with total nodal irradiation.


*Br. J. Cancer* (1980) **42**, 284

### PROGNOSTIC EFFECT OF EARLY DIAGNOSTIC SPLENECTOMY IN HODGKIN'S DISEASE: A RANDOMIZED TRIAL

J. ASKERGREN*, M. BJÖRKHOLM§, G. HOLM¶, B. JOHANSSON†, H. MELLSTEDT§, R. SUNDBLAD* AND G. SÖDERBERG‡

*From *The Department of Surgery, †Radiumhemmet and ‡The Department of Pathology, Karolinska Hospital, §The Department of Medicine, Seraphimer Hospital and ¶The Department of Clinical Immunology, Huddinge Hospital, Stockholm, Sweden*

Received 15 January 1980 Accepted 30 April 1980

**Summary.—A randomized trial is reported which evaluates the effect of early diagnostic splenectomy on the prognosis of patients with Hodgkin's disease (HD) and uncertain prognosis. This was started in January 1973 and concluded in April 1979. Sixty-seven patients were entered in the study and 31 were randomized for splenectomy. All patients except 2 received total nodal irradiation, excluding the splenic and hepatic areas. After 40 months' observation there was no difference between the groups in respect of survival and the number of recurrences. However, relapses occurred earlier in the splenectomized patients. Pneumococcal septicaemia was recorded in 2 splenectomized patients. It is concluded that prognosis is not improved by diagnostic splenectomy in HD patients with uncertain prognosis and treated with total nodal irradiation.**

EXPLORATORY LAPAROTOMY comprising diagnostic splenectomy with liver and lymphnode biopsy was introduced for the clinical staging of Hodgkin's disease by Glatstein *et al.* (1969). The rationale of this procedure has been supported by the unexpected finding of abdominal involvement in a large proportion of patients resulting in a more extensive treatment (Aisenberg *et al.*, 1971; Meeker *et al.*, 1972; Høst *et al.*, 1973; Rozman *et al.*, 1973; Kaplan *et al.*, 1973; Andersen & Videbaek, 1974; Smithers *et al.*, 1974; B.N.L.I., 1975; Irving, 1975; Somers *et al.*, 1976; Poulsen *et al.*, 1977; Lee *et al.*, 1978). Hitherto it has been accepted that early detection of advanced disease and the removal of involved spleens in HD should be beneficial to the course of the disease. However, early splenectomy in a group of 21 patients with HD treated with MOPP did not influence duration of survival or long-term remission when compared to historical matched non-splenectomized controls (Panettière *et al.*, 1977). No controlled studies are available to elucidate this question in less advanced HD.

This study was undertaken to evaluate the effect of early diagnostic splenectomy on prognosis in HD patients with uncertain prognosis, but without signs of splenic involvement.

### MATERIALS AND METHODS

*Patients.*—All previously untreated patients of 15–65 years of age admitted to Radiumhemmet from January 1973 to April 1979 were considered for the study. The diagnosis was established by lymphnode biopsy in all patients except 2 who had the diagnosis verified by aspiration biopsy (Björkholm *et al.*, 1977*a*). The Rye nomenclature was used for histological classification (Lukes *et al.*, 1966): lymphocytic predominance (LP), nodular sclerosis (NS), mixed cellularity (MC) and lymphocytic depletion (LD). All biopsy specimens were reviewed by the same pathologist (GS).

The clinical staging was based on a com-

Reprint requests to Dr Jutta Askergren, Department of Surgery, Danderyd Hospital, S-182 88 Danderyd, Sweden.

plete history, physical examination, liver-function tests, marrow biopsy, chest and plain abdominal X-rays, lymphangiography, and liver and spleen scans. The Ann Arbor nomenclature for clinical staging was used (Carbone *et al.*, 1971).

*Immunological tests.*—All untreated patients were tested. Lymphocytes were purified from defibrinated blood by gelatin sedimentation and treatment with carbonyl iron to remove phagocytic cells. A total lymphocyte count was made, and the lymphocyte DNA synthesis was determined as the incorporation of $^{14}C$-thymidine after 3 days' culture with mitogens (pokeweed mitogen, concanavalin A). The spontaneous DNA synthesis during the first 24 h of culture was also determined (Holm *et al.*, 1976). The results were expressed as the quotient:

$$\frac{\text{Experimental log ct/min}}{\text{Mean log ct/min of healthy controls}}$$

(20–35 years old) (Björkholm *et al.*, 1977*b*).

*Study protocol and treatment.*—The patients were divided into 3 groups according to prognosis: Favourable, Stage IA and IIA, LP and NS with right-sided presentation; Unfavourable, Stage IV, all histological groups and patients with enlarged spleens. Randomization for splenectomy was not considered to be justified in these 2 groups of patients. Only patients without clinical evidence of splenic involvement, in whom the prognosis was regarded as uncertain, were included in the study: Stage IA, IIA, LP and NS (left-sided presentation), IA, IIA, MC and LD, Stage IB, IIB and III, all histological groups. These patients received total nodal irradiation, excluding the splenic and hepatic areas, given with a 6MeV linear accelerator (Varian) with a mid-plane dose of 40–50 Gy. After the initial treatment, consisting of mantle field or inverted Y-field irradiation, depending on the first presentation of disease, the patients were stratified into 8 groups according to sex, age ($<35$ and $35\geqslant$) and histology (LP, NS and MC, LD), and were randomized for splenectomy or no splenectomy. After laparotomy the 2nd course of irradiation was started 8–10 weeks after the end of the initial treatment. The interval between the 2 courses of irradiation in the non-splenectomy group was 4–6 weeks. Patients who relapsed were treated with quadruple-drug combination chemotherapy (Björkholm *et al.*, 1977*a*).

*Surgical procedure.*—Laparotomy was performed through a left paramedian incision from the left costal margin to about 5 cm below the umbilical level. The incision allowed thorough inspection and palpation of the entire abdomen in order to map pathological lymph nodes. Biopsy samples were taken from the areas with lymphographic suspicion of involvement. In cases with negative lymphangiograms, but palpable iliac, para-aortic, coeliac, splenic or mesenteric nodes, biopsy samples were taken, and the sites were marked with metal clips. To avoid complications, large blind dissections were not performed. Before splenectomy the splenic artery was ligated above the pancreas through the lesser sac. Wedge liver biopsy specimens were taken from any suspected area or from the most accessible lobe.

In fertile women, the ovaries were moved and fixed to the back of the neck of the uterus and their lateral corners were marked with metal clips. Oophoropexy was also performed in all fertile women, except in 2 controls.

The spleens were cut in 5mm transverse sections and thoroughly inspected. Biopsy specimens from suspected areas were examined microscopically.

### RESULTS

### *The clinical material*

At the end of the study, in April 1979, 67 patients had been entered; 31 were randomized for splenectomy and 36 for no splenectomy. The 2 groups were comparable with regard to sex, age, observation time, lymphangiographic findings, clinical stage and histology (Tables I, II and III). As revealed in prospective studies, the immunological capacity evaluated by lymphocyte-function tests (see MATERIALS AND METHODS) is a strong prognostic predictor in HD (Björkholm *et al.*, 1977*a*). The 2 groups were also comparable with regard to their immune status (Table I).

The spleen was removed from 29 patients in the splenectomy group (Table II). Two patients in this group were not splenectomized; Case 19 refused surgery and inverted Y-field irradiation, but is still in complete remission; Case 31 died from gastric haemorrhage and generalized HD before splenectomy.

TABLE I.—*Characterization of the patient population*

| | Splen-ecto-mized | Non-splen-ecto-mized |
|---|---|---|
| Total population | 31 | 36 |
| Sex: F | 7 | 12 |
| M | 24 | 24 |
| Age (years): median | 32 | 32 |
| range | 15–64 | 15–65 |
| Obs. time from diagnosis (months): median | 40 | 41 |
| range | 4–75 | 3–75 |
| Clinical stage: I | 10 | 3 |
| II | 12 | 19 |
| III | 9 | 14 |
| Symptoms: A | 18 | 27 |
| B | 13 | 9 |
| Histology: LP | 5 | 3 |
| NS | 12 | 20 |
| MC | 11 | 12 |
| LD | 1 | 1 |
| Unclassified | 2 | 0 |
| Immunological tests | | |
| Total lymphocytes (log no./mm$^3$) | 3·09 | 3·13 |
| DNA synthesis (mean log quotient) | | |
| Spontaneous | 1·11 | 1·10 |
| Pokeweed mitogen (1 μg/ml) | 0·95 | 0·91 |
| Concanavalin A (20 μg/ml) | 0·95 | 0·94 |

In the non-splenectomy group (Table III) Case 51 was splenectomized on suspicion of progressive disease with rapidly increasing spleen size. His spleen was affected but was within the normal weight range. He refused inverted Y-field treatment, but is in complete remission 39 months after diagnosis. Case 52 was nephrectomized because of a hypernephroma. After 39 months he has no sign of relapse from HD or hypernephroma.

*Laparotomy findings*

In the splenectomy group spleens from 10 patients were involved (Table II). So also was Case 31, who died before operation but was found to have splenic HD at necropsy. In 2 patients, tumour was present in spleen and liver. Seven patients with positive lymphangiograms were treated with mantle-field irradiation before laparotomy. In 4 of them tumour was found in para-aortic nodes during surgery. On the other hand, 1/17 cases with negative lymphangiograms had HD in the biopsied abdominal lymph nodes. No patient with equivocal lymphangiograms had involvement of the biopsied abdominal nodes. All 4 patients with their first HD presentation in the inguinal lymph nodes had positive lymphangiograms. They received inverted Y-field irradiation before laparotomy. No lymphnode tumour was found during surgery. One of them had tumour involvement in the spleen.

After splenectomy the clinical stage was altered in 10 patients, 8 of them to a more advanced stage (Table II). Two patients with liver HD received quadruple-drug chemotherapy after surgery, as did patients in relapse.

*Surgical complications*

Early and late surgical complications were found in 4 patients (Table II).

*Infections*

Four severe infections were recorded, 3 in the splenectomy group and 1 in the non-splenectomy group (Tables II, III and IV). Case 21 developed generalized herpes zoster and pneumococcal septicaemia during chemotherapy, 6 months after splenectomy. Case 6 suffered a severe but nonfatal pneumococcal septicaemia 34 months after diagnosis. She was in complete remission after chemotherapy for a pulmonary recurrence. Case 30 died from generalized varicella during radiotherapy. In the non-splenectomy group Case 65 died of viral pneumonia. At necropsy no signs of HD were found.

*Prognosis*

The patients were evaluated at a follow-up in April 1979, 4–75 months after diagnosis. Relapse rates and patterns were similar in both groups (Tables II and III). However, the median time from diagnosis to relapse for the splenectomy group was

TABLE II.—*Clinical characteristics, laparotomy findings and course of disease in splenectomized patients*

| Case No. | Sex | Age (years) | Histology | Lymphography | Clinical stage | Laparotomy findings S | H | N | Post-op. stage | Clinical course | Period of observation (months) | Clinical condition at follow-up |
|---|---|---|---|---|---|---|---|---|---|---|---|---|
| 1 | M | 28 | NS (MC) | ± | II A | – | – | –* | II A | | 75 | CR |
| 2 | M | 20 | MC | – | I B | + | – | + | $III_{SN}B$ | | 70 | CR |
| 3 | M | 37 | NS (MC) | – | II A | – | – | – | II A | Ileus twice | 67 | CR |
| 4 | M | 48 | MC | + | III A | – | – | + | $III_{N}A$ | | 63 | CR |
| 5 | M | 30 | LP | ± | III A | – | – | – | II A | | 60 | CR |
| 6 | F | 28 | Unclass | – | II B | – | – | – | II B | Pulmonary relapse 24 months, sepsis 34 months | 58 | CR |
| 7 | M | 43 | NS | – | II B | + | – | – | $III_{S}B$ | Axillary relapse 20 months | 57 | CR |
| 8 | F | 25 | Unclass | – | I A | – | – | –* | I A | | 56 | CR |
| 9 | M | 28 | LP | – | I A | – | – | –* | I A | | 53 | CR |
| 10 | M | 59 | LP | – | I B | – | – | –* | I B | | 52 | CR |
| 11 | M | 41 | MC | + | II B | + | – | –‡ | $II_{S}B$ | | 48 | CR |
| 12 | M | 29 | LP | + | II A | – | – | –‡ | II A | | 47 | CR |
| 13 | F | 19 | NS (LD) | + | III B | – | – | – | II B | Left cervical relapse 31 months | 47 | CR |
| 14 | F | 32 | NS | – | II A | – | – | – | II A | | 47 | CR |
| 15 | M | 39 | NS (MC) | + | III B | – | – | –* | II B | | 40 | CR |
| 16 | M | 28 | NS (LP) | + | II A | – | – | –‡ | II A | | 40 | CR |
| 17 | M | 28 | MC | + | III A | + | – | + | $III_{SN}A$ | Ileus, hilar relapse 30 months | 39 | CR |
| 18 | M | 64 | MC | – | I A | + | – | – | $III_{S}A$ | | 39 | CR |
| 19 | M | 51 | MC | – | I A | refused operation | | | | | 37 | CR |
| 20 | F | 39 | MC | – | II A | + | – | – | $III_{S}A$ | | 28 | CR |
| 21 | M | 54 | MC | – | I B | + | + | – | $IV_{SH}B$ | Wound dehiscence 2 wks, sepsis 12 months | 24 | CR |
| 22 | M | 15 | NS(MC) | ± | III B | – | – | – | II B | Hilar relapse 12 months | 15 | PR |
| 23 | F | 22 | NS | – | II B | – | – | – | II B | | 14 | CR |
| 24 | M | 40 | LP | – | I A | + | – | – | $III_{S}A$ | Cicatric hernia | 14 | CR |
| 25 | F | 34 | MC | – | I A | – | – | – | I A | | 12 | CR |
| 26 | M | 23 | MC | + | III B | – | – | + | $III_{N}B$ | Cervical relapse 12 months | 42 | † |
| 27 | M | 28 | NS(MC) | + | III A | + | + | + | $IV_{SHN}A$ | Progress | 25 | † |
| 28 | M | 62 | NS(MC) | + | II A | – | – | –‡ | II A | Abdominal relapse 18 months | 24 | † |
| 29 | M | 44 | NS | – | II B | – | – | – | II B | Costal relapse 7 months | 18 | † |
| 30 | M | 19 | MC | – | I A | + | – | – | $III_{S}A$ | Generalized varicella 7 months | 7 | † |
| 31 | M | 60 | LD | + | III B | died before operation | | | | | 4 | † |

Lymphography: + positive; – negative; ± equivocal.

Laparotomy findings: + affected organ; – unaffected organ; * no palpable nodes, no biopsy; ‡ no palpable nodes, no biopsy, treated inverted Y-field irradiation before laparotomy; CR = complete remission; PR = partial remission; † = died.

TABLE III.—*Clinical characteristics and course of disease in non-splenectomized patients*

| Case No. | Sex | Age | Histology | Lymphography | Clinical stage | Clinical course | Period of observation (months) | Clinical condition at follow-up |
|---|---|---|---|---|---|---|---|---|
| 32 | M | 24 | LP | − | II A | | 75 | CR |
| 33 | F | 15 | NS | + | III A | Axillary relapse 40 months | 75 | CR |
| 34 | M | 63 | NS | + | III A | | 72 | CR |
| 35 | M | 32 | MC | + | III A | | 72 | CR |
| 36 | M | 23 | MC | ± | III A | | 71 | CR |
| 37 | M | 32 | NS | ± | III B | Axillary relapse 48 months | 70 | CR |
| 38 | M | 26 | NS | − | II A | Pulmonary relapse 24 months | 69 | CR |
| 39 | F | 24 | NS(LP) | − | II A | Inguinal relapse 29 months* | 68 | CR |
| 40 | M | 53 | NS(MC) | + | III A | | 66 | CR |
| 41 | F | 15 | LP | − | II A | | 65 | CR |
| 42 | M | 27 | MC | + | III A | | 65 | CR |
| 43 | M | 45 | LD | ± | II B | IV B, MOPP regimen | 63 | CR |
| 44 | M | 44 | MC | + | II A‡ | | 63 | CR |
| 45 | M | 47 | MC | − | II A | | 53 | CR |
| 46 | F | 23 | NS(MC) | − | I A | Cervical relapse 36 months | 53 | CR |
| 47 | F | 21 | NS | − | II B | | 53 | CR |
| 48 | M | 53 | MC | + | II A | | 52 | CR |
| 49 | F | 33 | NS(MC) | + | II A | Liver + spleens relapse 25 months | 41 | CR |
| 50 | M | 29 | NS(MC) | + | II A‡ | | 41 | CR |
| 51 | M | 37 | MC(LD) | − | I A | Growing spleen, splenectomized | 39 | CR |
| 52 | M | 37 | MC | + | III A | Hypernephroma, operation | 39 | CR |
| 53 | M | 22 | NS | + | III B | | 38 | CR |
| 54 | M | 21 | NS | + | III B | Cervical relapse 28 months | 34 | CR |
| 55 | M | 19 | NS | − | II A | | 24 | CR |
| 56 | M | 62 | NS | − | II B‡ | | 22 | CR |
| 57 | M | 50 | LP | + | II A‡ | Hypersplenism 14 months Splenectomized 20 months | 22 | CR |
| 58 | F | 17 | NS | − | II A | | 20 | CR |
| 59 | M | 65 | NS | − | I A‡ | | 19 | CR |
| 60 | F | 43 | NS(MC) | + | III A | | 15 | CR |
| 61 | M | 32 | NS(LP) | − | III A | | 9 | CR |
| 62 | F | 33 | NS | − | II B | | 3 | CR |
| 63 | F | 54 | NS(MC) | − | II A | Progress spleen uninvolved at necropsy | 28 | † |
| 64 | F | 50 | MC | + | III A | Histiocytic lymphoma at necropsy | 21 | † |
| 65 | F | 30 | MC | + | III B | Viral pneumonia NED at necropsy | 18 | † |
| 66 | M | 51 | NS | + | III B | Progress, bleeding ulcer | 8 | † |
| 67 | M | 57 | MC | − | II A | Progress, brain metastases | 8 | † |

* Patient refused oophoropexia, only para-aortic field.
† Died.
‡ Inverted Y-field treatment before mantle field.
CR = complete remission.

TABLE IV.—*Clinical course*

| | Splen-ecto-mized | Non-splen-ecto-mized |
|---|---|---|
| Total number of patients | 31 | 36 |
| Relapses | 8 | 8 |
| Time from diagnosis to relapse (months) | | |
| Median | 19·0 | 28·5 |
| Range | 7–31 | 14–48 |
| Bacterial septicaemia | 2 | 0 |
| Lethal viral infection | 1 | 1 |
| Deaths, total | 6 | 5 |

FIG. 1.—Actuarial survival according to splenectomy (31) or non-splenectomy (36)

19·0 months compared to 28·5 months in the non-splenectomy group ($P < 0{\cdot}05$, Wilcoxon's test).

In the splenectomy group 6 patients have died (Table II) compared with 5 deaths in the non-splenectomy group (Table III). The diagnosis in Case 64 was reassessed at necropsy to histiocytic lymphoma. One patient in each group died from viral infections without evidence of HD. The survival determined according to Cutler & Ederer (1958) revealed no difference between the groups (Fig. 1; Björkholm *et al.*, 1977*a*).

### DISCUSSION

The present study comprises 67 patients, 31 of whom were randomized for diagnostic splenectomy. In a follow-up concluded in April 1979, there was no difference between the groups with regard to mortality and time of survival. Six patients have died in the splenectomy group and 5 in the non-splenectomy group. Viral infections were the cause of death in 1 patient in each group. The others died from the disease, except Case 64 (see above). There was no difference in relapse frequency between the 2 groups, but relapses seemed to occur earlier among the splenectomized patients. Splenectomy as a therapeutic adjunct seems little justified, particularly considering the serious nature of the bacterial septicaemia in 2 splenectomized cases (Chilcote *et al.*, 1976; Weitzman & Aisenberg, 1977).

Splenic involvement was seen in 11/30 cases, which is comparable with the 10–40% reported in other series (Kaplan *et al.*, 1973; Lee *et al.*, 1978; Cannon *et al.*, 1974). On the basis of this figure, it is highly unlikely that the spleens were uninvolved in all control patients. It should also be noted that the splenic area was not included in the irradiation field. Thus, splenic recurrences would have been expected in some patients in the control group. However, splenic involvement was only noted in 3/8 recurrences in the control group. In one patient with initial clinical Stage III A and a positive lymphangiogram, a liver relapse was suspected. As liver involvement is almost always associated with splenic involvement this may be a recurrence caused by persisting HD in the spleen. Another patient showed signs of hypersplenism 14 months after diagnosis. He was successfully splenectomized, and has no signs of active disease. Finally the 3rd patient in the control group who had splenic involvement was splenectomized after his mantle-field treatment on suspicion of splenic involvement. The incidence of symptomatic splenic recurrences in the non-splenectomized group (3/36) is significantly lower than that of splenic HD in the splenectomized patients (11/30; $P < 0.001$, Fisher's exact test).

The reason so few splenic relapses have appeared is as yet unknown. One possibility is that splenic HD may heal in the absence of disease in other locations. This might parallel the rare occurrence of tumour metastases in the spleen (McLure & Hwa Park, 1975). Alternatively, the histopathological picture in HD spleens may be a sign of a reactive rather than a malignant process (Amiel & Droz, 1978). As a 3rd alternative, splenic recurrences in non-splenectomized patients may occur late in the disease, thereby escaping detection during the period of observation. However, the recurrences in the splenectomized group were considerably earlier than those in the non-splenectomized group (Tables II and III). This is not likely to depend on the longer interval between the mantle and abdominal irradiation in the splenectomized patients, as 6/8 recurrences occurred in the first irradiated area. Moreover, the routine splenectomizing of HD patients before irradiation might favour tumour growth.

It may be concluded that early diagnostic splenectomy in HD under the treatment protocol used in this study does not improve prognosis. Rather, it may lead to surgical complications, increased risk of severe infections and may be associated with earlier relapse of disease.

We are indebted to Dr Stephan Ogenstad, Department of Statistics, University of Stockholm, for making the statistical analysis, James Ware, M.A., M.D., F.R.C.S., for reviewing and Ms Monica Blixth for typing the manuscript.

This work was supported by grants from the Swedish Cancer Society and the Stockholm County Council.

### REFERENCES

AISENBERG, A. C., GOLDMAN, J., RAKER, J. & WANG, C. (1971) Spleen involvement at the onset of Hodgkin's disease. *Ann. Intern. Med.*, **74**, 544.

AMIEL, J. L. & DROZ, J. P. (1978) Staging and treatment of Hodgkin's disease. *Eur. J. Cancer*, **14**, 1.

ANDERSEN, E. & VIDEBAEK, A. A. (1974) Diagnostic laparotomy in Hodgkin's disease. *Scand. J. Haematol.*, **12**, 5.

BJÖRKHOLM, M., HOLM, G., MELLSTEDT, H., JOHANSSON, B., ASKERGREN, J. & SÖDERBERG, G. (1977*a*) Prognostic factors in Hodgkin's disease. I. Analysis of histopathology, stage distribution and results of therapy. *Scand. J. Haematol.*, **19**, 487.

BJÖRKHOLM, M., HOLM, G. & MELLSTEDT, H. (1977*b*) Persisting lymphocyte deficiencies during remission in Hodgkin's disease. *Clin. Exp. Immunol.*, **28**, 389.

BRITISH NATIONAL LYMPHOMA INVESTIGATION (1975) The value of laparotomy and splenectomy in the management of early Hodgkin's disease. *Clin. Radiol.*, **26**, 151.

CANNON, W. B., KAPLAN, H. S., DORFMAN, R. F. & NELSEN, T. S. (1974) Staging laparotomy with splenectomy in Hodgkin's disease. *Surg. Ann.*, **7**, 103.

CARBONE, P. P., KAPLAN, H. S., MUSSHOFF, K., SMITHERS, D. W. & TUBIANA, M. (1971) Report of the committee on Hodgkin's disease staging classification. *Cancer Res.*, **31**, 1860.

CHILCOTE, R., BAEHNER, R. & HAMMOND, E. D. (1976) Septicemia and meningitis in children splenectomized for Hodgkin's disease. *N. Engl. J. Med.*, **295**, 798.

CUTLER, J. S. & EDERER, F. (1958) Maximum utilization of the life table method in analysing survival. *J. Chronic Dis.*, **8**, 699.

GLATSTEIN, E., GUERNSEY, J. M., ROSENBERG, S. A. & KAPLAN, H. S. (1969) The value of laparotomy and splenectomy in the staging of Hodgkin's disease. *Cancer*, **24**, 709.

HOLM, G., MELLSTEDT, H., BJÖRKHOLM, M. & 4 others (1976) Lymphocyte abnormalities in untreated patients with Hodgkin's disease. *Cancer*, **37**, 751.

HØST, H., ABRAHAMSEN, A. F., JÖRGENSEN, O. G. & NORMANN, T. (1973) Laparotomy and splenectomy in the management of Hodgkin's disease. *Scand. J. Haematol.*, **10**, 327.

IRVING, M. (1975) The role of surgery in the management of Hodgkin's disease. *Br. J. Surg.*, **62**, 853.

KAPLAN, H. S., DORFMAN, R. F., NELSEN, T. S. & ROSENBERG, S. A. (1973) Staging laparotomy and splenectomy in Hodgkin's disease: Analysis of indications and patterns of involvement in 285 consecutive unselected cases. *Natl Cancer Inst. Monogr.*, **36**, 291.

LEE, Y. T., LUKES, R., FINCK, E., FEINSTEIN, D. & POWARS, D. (1978) Staging laparotomy and splenectomy for Hodgkin's disease. *Am. Surg.*, **44**, 215.

LUKES, R. J., CRAVER, L. F., HALL, T. C., RAPPAPORT, H. & RUBEN, P. (1966) Report of the nomenclature committee. *Cancer Res.*, **26**, 1311.

MEEKER, W. R., RICHARDSON, J., WEST, W. & PARKER, J. (1972) Critical evaluation of laparotomy and splenectomy in Hodgkin's disease. *Arch. Surg.*, **105**, 222.

MCLURE, J., JR & HWA PARK, Y. (1975) Solitary metastatic carcinoma of the spleen. *South. Med. J.*, **68**, 101.

PANETTIÈRE, F. J., COLTMAN, C. A. JR & DELANEY, F. C. (1977) Splenectomy, chemotherapy and survival in Hodgkin's disease. *Arch. Intern. Med.*, **137**, 341.

POULSEN, H., BENGMARK, S., BÖRJESSON, B., FLODGREN, P., KALLUM, B. & LANDBERG, T.

(1977) Staging laparotomy with splenectomy in Hodgkin's disease. *Acta Chir. Scand.*, **143**, 347.

ROZMAN, C., TRIGINER, J., RIBAS-MUNDO, M., FERRAN, C., VISA, J. & GONZALES, E. (1973) The value of laparotomy and splenectomy in the staging of 56 patients with Hodgkin's disease. *Acta Haematol.*, **50**, 321.

SMITHERS, D. W., LILLICRAP, S. C. & BARNES, A. (1974) Patterns of lymph node involvement in relation to hypotheses about the modes of spread of Hodgkin's disease. *Cancer*, **34**, 1779.

SOMERS, R., BURGERS, J., HART, A. & VAN COEVERDEN, S. (1976) Laparotomy in Hodgkin's disease: Incidence of complications, significance of splenic involvement. *Neth. J. Med.*, **19**, 219.

WEITZMAN, S. & AISENBERG, A. (1977) Fulminant sepsis after successful treatment of Hodgkin's disease. *Am. J. Med.*, **62**, 47.